# A New Barrier to Dispersal Trapped Old Genetic Clines That Escaped the Easter Microplate Tension Zone of the Pacific Vent Mussels

**DOI:** 10.1371/journal.pone.0081555

**Published:** 2013-12-02

**Authors:** Sophie Plouviez, Baptiste Faure, Dominique Le Guen, François H. Lallier, Nicolas Bierne, Didier Jollivet

**Affiliations:** 1 Université Pierre et Marie Curie-Paris 6, Laboratoire Adaptation et Diversité en Milieu Marin, Station Biologique de Roscoff, Roscoff, France; 2 CNRS UMR 7144, Station Biologique de Roscoff, Roscoff, France; 3 Division of Marine Science and Conservation, Nicholas School of the Environment, Duke University, Beaufort, North Carolina, United States of America; 4 Université Montpellier 2, Montpellier, France; 5 CNRS UMR 5554, Institut des Sciences de l’Evolution, Station Méditerranéenne de l’Environnement Littoral, Sète, France; Australian Museum, Australia

## Abstract

Comparative phylogeography of deep-sea hydrothermal vent species has uncovered several genetic breaks between populations inhabiting northern and southern latitudes of the East Pacific Rise. However, the geographic width and position of genetic clines are variable among species. In this report, we further characterize the position and strength of barriers to gene flow between populations of the deep-sea vent mussel *Bathymodiolus thermophilus*. Eight allozyme loci and DNA sequences of four nuclear genes were added to previously published sequences of the cytochrome *c* oxidase subunit I gene. Our data confirm the presence of two barriers to gene flow, one located at the Easter Microplate (between 21°33′S and 31°S) recently described as a hybrid zone, and the second positioned between 7°25′S and 14°S with each affecting different loci. Coalescence analysis indicates a single vicariant event at the origin of divergence between clades for all nuclear loci, although the clines are now spatially discordant. We thus hypothesize that the Easter Microplate barrier has recently been relaxed after a long period of isolation and that some genetic clines have escaped the barrier and moved northward where they have subsequently been trapped by a reinforcing barrier to gene flow between 7°25′S and 14°S.

## Introduction

Genetic structure is easier to detect and understand in a one-dimensional system than in a two-dimension space [1]. However, even in a one-dimension space, detecting a genetic cline with a correlation between genetic differentiation and geographical distance does not always mean that populations are following an isolation-by-distance (IBD) model. Such a correlation can also be due to the presence of barriers to dispersal (e.g., [2]) or secondary contacts between previously isolated populations under expansion (e.g., [3]).

When detecting a genetic cline, one should consider the possibility that the location of this cline may be due to the presence of a natural barrier to dispersal because clines are expected to be trapped by such a barrier [4], [5]. Genetic clines depend on the relative impact of dispersal, selection (e.g., [6], [7]) and the recent demographic history of populations (e.g., [8]), and a natural barrier to dispersal impacts the balance among these parameters. As a result, clines often typify adaptive gradients [9], [10] or hybrid zones (i.e., regions containing recombinant individuals between genetically differentiated populations).

The geographic region in which a balance between dispersal and selection is maintained is defined as a tension zone. Tension zones tend to stabilize over natural barriers to dispersal [5], [7], [11], maintaining a cline around that barrier. They can also couple with a local adaptation cline and be stabilized at an environmental boundary [12]. If the origin of stabilized clines is difficult to establish from a single gene (e.g., isolation-by-distance without natural barriers to dispersal, hybrid zone), comparing allele frequencies between genes and gene divergences can help explain its emergence and, if a barrier is present, to identify its position more precisely.

Deep-sea hydrothermal vents are patchily distributed along mid-ocean ridges and back-arc basins. Along the East Pacific Rise (EPR), they follow a one-dimension pattern, ideal for testing an isolation-by-distance model of populations [13]. However, vent displacements along the ridge and eruptive phases leading to local faunal extinctions together with transform faults which likely impede gene flow are able to seriously alter expectations of such population models [14].

Comparative phylogeographic analyses of deep-sea hydrothermal vent species have previously shown the presence of a genetic break between the northern and southern regions of the EPR [15], [16], [17]. These studies established a shared vicariant event among species and suggested the emergence of a barrier to dispersal near the equator about 1.5 to 2 Mya [18]. However, the width and position of the barrier was not matching among species: some such as the tube-dwelling polychaete *Alvinella pompejana* displayed an abrupt separation of the populations across the equator [3] whereas others showed genetic patterns closer to an isolation-by-distance model [15]. Interestingly, the deep-sea mussel *Bathymodiolus thermophilus* exhibited a smooth clinal distribution of mitochondrial lineages along the EPR (13°N to 21°33′S) [15], [17] and the presence of a cryptic species, *B.* aff. *thermophilus* further south (31°S–32°S) [15].

*Bathymodiolus* species have been supposed to be long-distance dispersers because of their planktotrophic mode of larval development [19] and the possibility of larvae reaching the upper (photic) layers of the water column [20]). The wide-dispersal capabilities of *Bathymodiolus* have been confirmed in several population genetic studies showing the absence of genetic differentiation across the Atlantic [21] and across the Gulf of Mexico (Mississippi Canyon and Alaminos Canyon, 550 km apart) [22]. Such dispersal characteristics could strongly favour population connectivity among geographically isolated sites. However, hybrid zones resulting in an abrupt change of allele frequencies over relatively short distances have also been observed in both the Atlantic [23] and the Pacific [24].

The likely cause of the clines observed at the EPR has been a source of debate. Although Plouviez *et al.* [17] suggested the observed genetic shifts might be the consequence of a natural barrier to dispersal that separated two interacting *Bathymodiolus* units, Audzijonyte & Vrijenhoek [25] proposed that the differentiation observed could simply be obtained under IBD. However, Johnson *et al.* [24] recently refuted IBD by describing a tension zone localised at the Easter Microplate between *B. thermophilus* and *B.* aff. *thermophilus* (renamed *B. antarcticus* by the authors). One of the loci, S-Adenosyl Homocysteine Hydrolase (SAHH), displayed a discordant cline position with fixed substitutions around the geographic zone previously identified by Plouviez *et al.* [17] to be a barrier to gene flow. The small likelihood of discovering fixed alleles over such a small spatial scale under IBD, and the fact that mitochondrial DNA exhibited an abrupt shift in allele frequency at the exact same position [17] prompted a reinvestigation of the hypothesis of a second barrier to gene flow.

In the present study, we obtained allozyme and DNA sequence datasets for four nuclear genes including the SAHH marker and analysed this new dataset together with mitochondrial haplotypes previously obtained from the EPR mussels to further investigate how genetic diversity is structured across the EPR and more specifically to test for the homogeneity of gene divergences across identified barriers. We obtained evidence for the existence of two barriers, including the one positioned at 7°25′S–14°S. We thus propose that a pair of (semi-)permeable barriers along the EPR is likely responsible for the geographically discordant clinal distribution of alleles in this region.

## Materials and Methods

### Ethics statement

No specific permits were required to perform field studies described in this article. No specific permissions were required to access geographic localities and sample specimens (sampling sites belong to international waters). The locations are not privately owned or protected in any way. The field studies did not involve endangered or protected species.

### Collection

*Bathymodiolus thermophilus* specimens were sampled from seven deep-sea hydrothermal vent fields along the East Pacific Rise (EPR) from 9°50′N to 21°33′S (Table 1) using the tele-manipulated arm of the manned submersible Nautile operated from the oceanographic vessels Le Nadir and L’Atalante during three oceanographic cruises: at 9°50′N during HOT 1996 and Mescal 2010 and from latitudes 7°25′S to 21°33′S during BIOSPEEDO 2004. During the three cruises, all fresh specimens were measured and dissected on board and tissues (mantle and muscle) were preserved in 80% alcohol. In addition, the anterior muscle of each individual was also frozen in liquid nitrogen for allozyme analyses during BIOSPEEDO 2004 and Mescal 2010.

**Table 1:** Location and sample size of populations sampled for allozymes (n_allo_), nuclear genes (n_SAHH_, n_Lyso_, n_Sulfo1_, n_EF1α_) and mitochondrial (n_mtCOI_) gene.

Locality	Site name	Latitude	Depth	n_allo_	n_Sulfo1_	n_Lyso_	n_SAHH_	n_EF1α_	n_mtCOI_*
		Longitude							
GR									
1°N	Mussel Bed	0°48′ N	2486	-	-	-	-	-	12
		86°09′ W							
1°N	Rose Garden	0°48′ N	2460	-	-	-	-	-	12
		86°14′ W							
EPR									
13°N	-	12°48′ N	2630	-	-	-	-	-	12
		103°56′ W							
11°N	-	11°25′ N	2515	-	-	-	-	-	12
		103°47′ W							
9°50′N ^‡^	East Wall	9°50′ N	2530	66	9	11	6	4	45
		104°17′ W							
7°25′S ^‡^	Last Hope	7°25′ S	2735	9	16	19	25	25	48
		107°47′ W							
11°S	-	11°18′ S	2669	-	-	-	-	-	12
		110°32′ W							
14°S ^‡^	Lucky Eric	13°59′ S	2623	38	12	14	12	-	30
		112°29′ W							
17°25′S ^‡^	Oasis	17°25′ S	2575	69	12	10	11	-	21
		113°12′ W							
17°35′S ^‡^	Ms Wormwood	17°35′ S	2595	23	12	15	10	-	60
		113°15′ W							
18°33′S ^‡^	Animal Farm	18°33′ S	2636	33	12	12	11	-	30
		113°24′ W							
21°33′S ^‡^	Gromit	21°33′ S	2800	108	8	14	19	14	27
		114°18′ W							
PAR									
31°S	-	31°09′ S	2332	-	-	-	-	-	12
		111°55′ W							
32°S	-	31°51′ S	2331	-	-	-	-	-	12
		112°02′ W							
38°S	Foundation hotspot	37°70′ S	2200	-	2	2	2	2	2
		110°87′ W							

GR, Galapagos Rift; EPR, East Pacific Rise; PAR, Pacific Antarctic Rise. Depth is given meters. Sulfo 1, Sulfotransferase paralogue 1; Lyso, Lysozyme; SAHH, S-Adenosyl Homocysteine Hydrolase; EF1α, Elongation Factor 1α; mtCOI, cytochrome oxidase I. n_SAHH_, n_Lyso_, n_Sulfo1_, n_EF1α_, total number of recaptured individuals for each nuclear gene. *, sequences from [15], [17]. ^‡^, populations used for the Monmonier analysis.

### Allozyme genotyping

Eight enzyme loci were genotyped for each individual of *B. thermophilus* collected from 7°25′S to 21°33′S (Table 1) following the protocols of Boutet *et al* [26]: Phosphoglucomutase (*Pgm*, E.C. 5.4.2.2), Mannose phosphate isomerase (*Mpi*, E.C. 5.3.1.8), Octopine deshydrogenase (*Odh*, 1.5.1.11), Leucine amino peptidase *(Lap*, 3.4.11.1), Glucose phosphate isomerase (*Gpi,* 5.3.1.9), Malate deshydrogenase-1 and -2 (*Mdh 1* and *2*, 1.1.1.37), and Hexokinase-1 (*Hk*, 2.7.1.1). Alleles were numbered according to their relative mobility from the most frequent allele (labelled as 100) previously determined for the Atlantic species *B. azoricus*, this species was used as a reference (see [26]).

The program Genetix 4.05.2 [27] was used to perform population genetic analyses on allozyme data. For each locus, allele frequencies, heterozygosities and Weir & Cockerham [28] *f* statistic (departure from Hardy-Weinberg equilibrium tested by a 1000-permutations test) were estimated for each population along the EPR. The overall genetic differentiation across populations was estimated at all loci using Weir & Cockerham's θ estimator [28] and 1000 permutations used to determine significance. The isolation-by-distance model was tested with a Mantel Spearman test with 5000 permutations using Genepop 4.0.10 [29].

### DNA sequencing

Genomic DNA was extracted using a CTAB-PVP extraction procedure following Jolly *et al.* [30]. Mitochondrial lineages of *B. thermophilus* were identified from cytochrome oxidase I gene (mtCOI) sequences previously obtained by Plouviez *et al.* ([17], Table 1). Sequences from three nuclear genes (GenBank accession numbers KC858658- KC858846, see Table S1) were obtained from the same individuals using the mark-recapture (MR) cloning technique developed by Bierne *et al.* [31] with two times the capture effort. MR-cloning was chosen over direct sequencing of PCR products because of the presence of insertions/deletions in intronic regions and to have access to linkage disequilibrium among polymorphic sites without the use of computer algorithms to determine allelic phase. Primers developed by Faure *et al.* [23] were used for amplification of introns in the S-Adenosyl Homocysteine Hydrolase (SAHH) and Lysozyme (Lyso) genes, previously obtained from EST sequences from a *Bathymodiolus azoricus* cDNA library. The SAHH gene contains a poly-A tract varying in length among individuals, the length polymorphism in this region was not included in the analyses due to the high potential error rate resulting from PCR and cloning. Specific primers for the Sulfotransferase (Sulfo) gene were also designed from the cDNA sequence data (BtSulfo-F: 5′-TCTTTAAAGTCAGGATCACATTGG-3′, BtSulfo-R: 5′-TAAGGCAAAGTGGAACAACGAGACCGC-3′). Sequences from Sulfotransferase were sorted in two paralogous genes called Sulfo1 and Sulfo2 from individual allele recaptures, respectively. Because of the low number of sequences recaptured from Sulfo2, only Sulfo1 sequences were used in this study.

Faure *et al.* [23] used a similar MR-cloning approach on two individuals from 37°70′S for the three nuclear genes studied in the present paper. Faure *et al.* [32] also used a MR-cloning approach for a fourth nuclear gene (Elongation Factor 1α, EF1α), for which four of our populations were sampled and already sequenced prior to this population analysis. This gene was thus included in our analyses. In Johnson *et al.* [24], a study done in parallel to ours, the SAHH and EF1α genes were also used but alleles were obtained by direct sequencing. Because of the presence of insertion/deletion in the intronic region, Johnson *et al.* [24] were able to analyze only a quarter of the sequence length we obtained by MR-cloning. Consequently, this did not allow us to include these new datasets into our SAHH and EF1α analyses.

DNA sequences obtained from the MR-cloning method were visualized and edited using CodonCode Aligner 2.0.6 (http://www.codoncode.com/aligner/). Sequence alignments were initially performed with ClustalW [33] and improved manually. The number of individuals for which at least one of the two alleles was recaptured (number of recaptured individuals) varied from one population to another is indicated in Table 1. Because of the random nature of the recapture, it was not possible to distinguish ‘true’ homozygotes from heterozygotes with the recapture effort. Consequently, and because our main results is based on the coalescence theory, only the most recaptured allele was retained from each individual to avoid sample bias when performing demographic analyses and genetic diversity estimations (see: Table 1). Multiple recaptures allowed us to discard intra-individual *in vitro* recombinants and putative artefactual/somatic mutations. Recombinants between different individuals (1–2% of the dataset for each population) from the same PCR set were detected (and removed) based on abnormal combinations of the 5′-tails.

### Polymorphism and divergence from DNA sequences

For each locus and locality, nucleotide diversity (*π_n_*) and Watterson's theta (*θ_w_*) were estimated using DnaSP 4.10.3 [34]. Phylogenetic relationships among alleles were estimated using the median joining algorithm of the Network software (version 4.5.0.0; www.fluxus-engineering.com) [35] to detect potentially divergent clades. The geographic distribution of divergent clades was then examined to locate potential barriers to gene flow by plotting synthetic clade-specific allele frequency distributions for each locality. To test for gene divergence homogeneity across barriers, divergence time between clades was estimated using the formula T = D/2r) under the assumption of a local molecular clock (tested using the BEAUti/BEAST 1.4.8 package [36] with parameters previously described in [17]), where D is the average net divergence between geographic clades and r the mutation rate per site per million years [37]. The initial separation between *B. thermophilus* and *B. azoricus* (across the Isthmus of Panama, set to about 8–12 Mya for deep-sea fauna; [38]) was used as calibration point using a published dataset [23]. This calibration of deep-sea fauna separation across the Isthmus of Panama has been used successfully in other species [39].

Neutrality of loci was also tested using a multi-loci Hudson-Kreitman-Aguadé test (HKA, [40]) performed separately for each divergent clade, with one sequence of *B. azoricus* as an outgroup, via the software HKA (J. Hey's web page: http://lifesci.rutgers.edu/~heylab/HeylabSoftware.htm#HKA). This test compares polymorphism and divergence at several loci to detect if at least one of these loci displays a departure from neutral evolution. This test was preferred to the McDonald-Kreitman [41] test because of the absence of fixed non-synonymous mutations between the two deep-sea mussel species.

### Statistical evidence for a barrier to gene flow at 7°25′S-14°S

A Monmonier algorithm was implemented using Barrier 2.2 [42] that compares matrices of multigene genetic distances (F-statistics, *φ_st_*, estimated with DnaSP 4.10.3 [34]) and geographic distances under the assumption of gene flow breaks. This Bayesian program was used to determine the geographic position of a potential barrier along the East Pacific Rise (insertions/deletions were coded as presence/absence). As the software Barrier 2.2 does not hold missing *φ_st_* values in the matrix, the EF1α gene and some of the populations for which, at least one gene was missing were discarded from the analysis (see Table 1). The 38°S population was not included because of its small sample size. Localities from each side of the 7°25′S–14°S barrier were grouped together to test for genetic differentiation across this barrier using *φ_st_* [43] computed using DnaSP 4.10.3 (1000-permutations [34]).

### Migration rates and demographic history of populations

Migration rates across the 7°25′S–14°S barrier and the effective size of the southern and northern populations were estimated by fitting an isolation with migration model (IMa2 program, [44]) using previously described parameters [3], with the following exceptions: upper bounds of uniform priors set at θ = 50 (population size), m = 5 (migration rate) and t = 30 (divergence time). Demographic and migration parameters were calibrated using divergence across Panama to inform mutation rates of loci (geometric mean among loci following a strict molecular clock) and a generation time of 2 years as previously estimated by Faure *et al.* [23].

### Hybrid/introgressed individuals detected using SAHH RFLP analysis

A *Hinf I* restriction site polymorphism fixed between the two main sets of SAHH alleles was identified and used to check for the occurrence of putative introgressed/hybrid individuals between mitochondrial lineages by looking for individuals that possess one SAHH allele from each of the two distinct clades (called N/S individuals). An RFLP analysis was then performed using the *Hinf I* site to detect N/S individuals by screening all individuals from the *Bathymodiolus* collection. Incubation of PCR-products was done at 37°C for 1.5 hour in a 20 μl total volume containing 17 μl of PCR product, 1X buffer (supplied by the manufacturer) and 10 U of *Hinf I* (Ozyme^™^).

## Results

### Geographic distribution of allozymes

**Figure 1:**
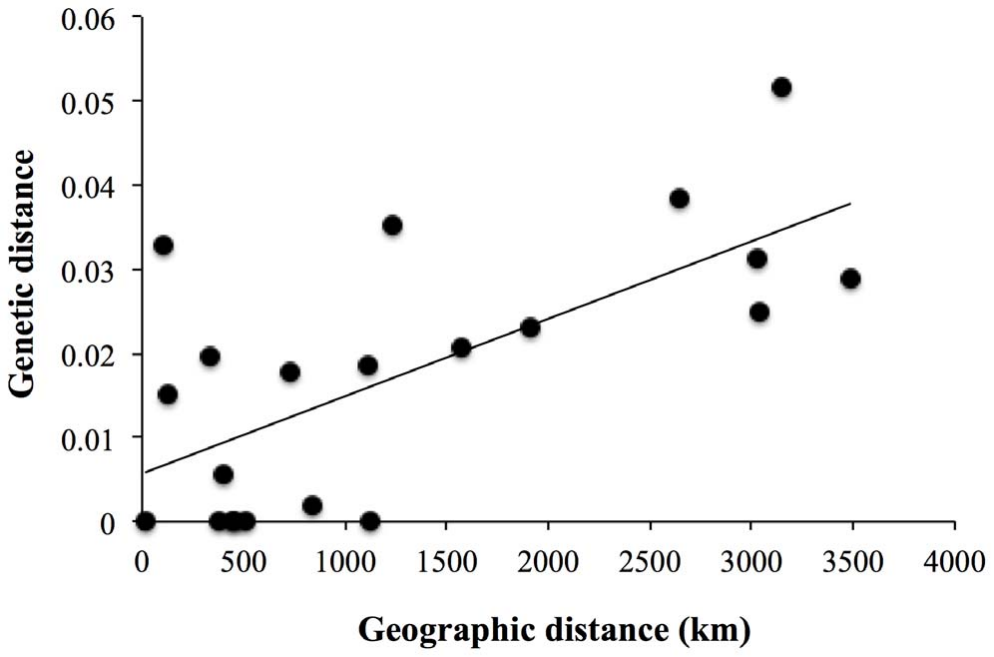
Relationship between pairwise genetic distances (θ/(1-θ)) from allozymes and geographic distances (in kilometres).

A series of differentiation tests were performed on *B. thermophilus* populations located along the EPR. Among the eight allozyme loci, four (*Mpi*, *Odh*, *Mdh 2* and *Hk)* were nearly monomorphic with the most frequent allele occurring at a frequency greater than 95% in all populations. The remaining four loci (*Pgm*, *Lap*, *Gpi* and *Mdh 1*) exhibited enough polymorphism to investigate their allelic distribution over the range of the EPR. Genetic differentiation was very low and not statistically significant overall (θ = 0.016, P value > 0.05), showing the absence of any strong genetic structure at allozyme loci along this portion of the EPR. The Mantel Spearman test showed a slight but significant correlation (P value = 0.05) between the genetic distance (θ/(1-θ)) and the geographic distance between vent fields (Fig. 1).

### Polymorphism and divergence from DNA sequences

**Figure 2:**
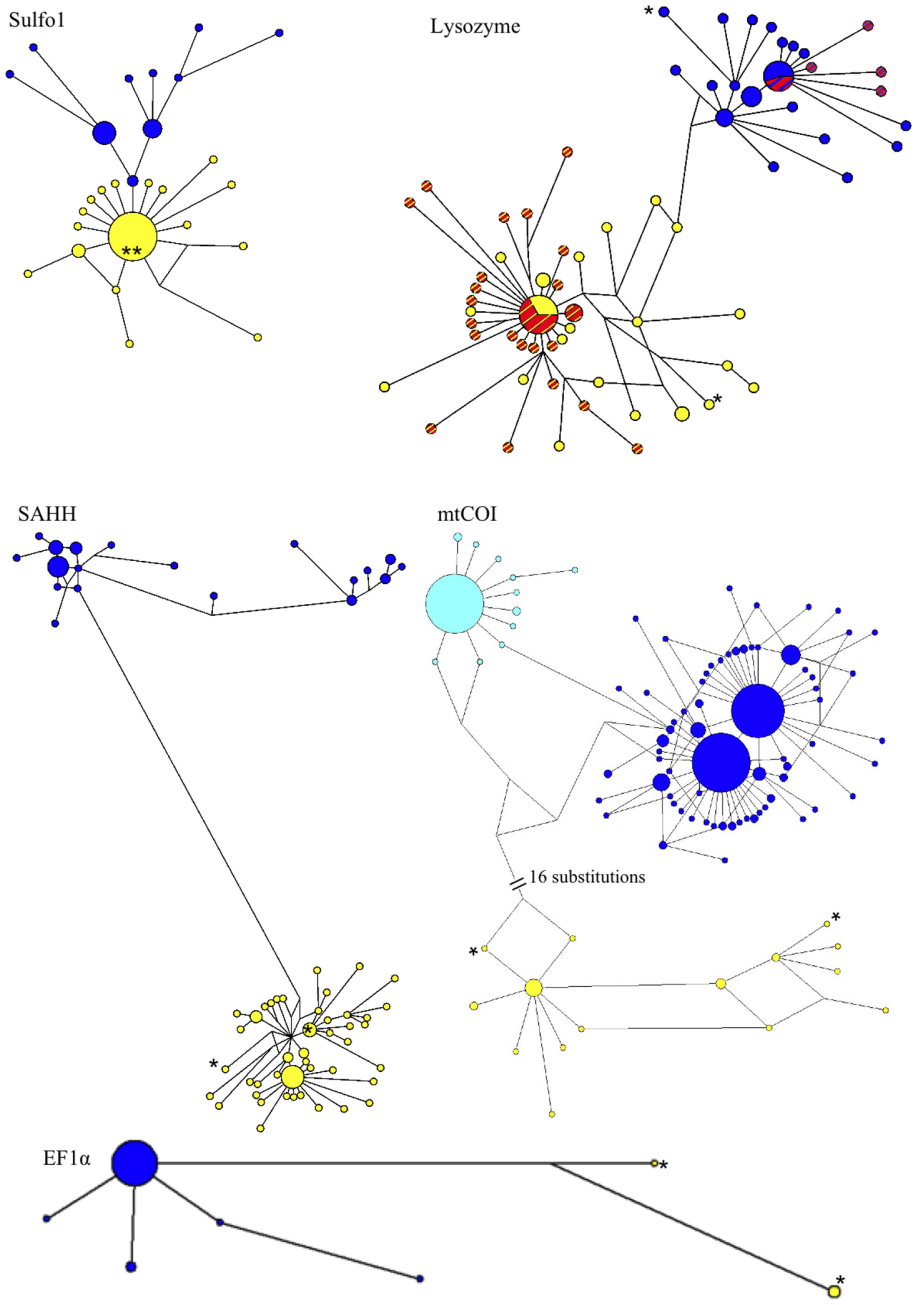
Median Joining Networks on the three nuclear genes and the mitochondrial cytochrome oxidase I gene. For each gene, the sizes of haplotype/allele circles and lengths of connecting lines are proportional to the number of individuals and the number of mutations that separate two linked haplotypes/alleles, respectively (length is not reflected in the 16-substitution link indicated on the mtCOI network). Colours represent divergent clades used for mapping the geographic distribution of alleles in Figure 3. For the nuclear genes, dark blue circles correspond to clades 1 and yellow circles to clades 2 in the manuscript. For mtCOI, light blue circles = clade 1, dark blue circles = clade 2, yellow circles = clade 3. For the Lysozyme gene, position of the 1-bp deletion in the network is represented by red stripes within both the yellow and the blue circles. *, position of individuals from 38°S in the network. Sulfo 1, Sulfotransferase paralogue 1; Lyso, Lysozyme; SAHH, S-Adenosyl Homocysteine Hydrolase; EF1α, Elongation Factor 1α; mtCOI, cytochrome oxidase I.

**Figure 3:**
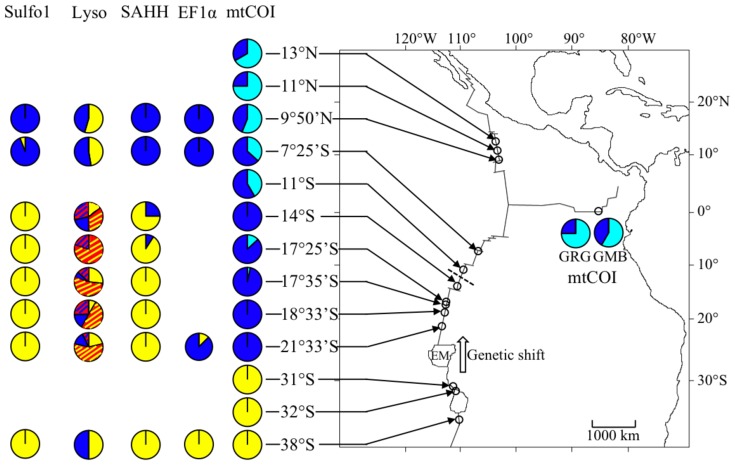
Geographic distribution of divergent alleles for three nuclear genes and the mitochondrial cytochrome oxidase I gene. Colours match divergent clades identified in Figure 2. Stripes indicate the presence of the “deletion” in the Lysozyme gene on either the yellow clade (red stripes on yellow background) or the blue clade (red stripes on blue background). The dashed line depicts the recent barrier to gene flow identified by the Monmonier analysis. The white-block arrow represents the hypothesized northward genetic shift of the tension zone for some genes. Sulfo 1, Sulfotransferase paralogue 1; Lyso, Lysozyme; SAHH, S-Adenosyl Homocysteine Hydrolase; EF1α, Elongation Factor 1α; mtCOI, cytochrome oxidase I; EM, Easter Microplate; GRG, Galapagos Rose Garden; GMB, Galapagos Mussel Bed.

Population structure across the Easter Microplate was found for two genes (Fig. 2, 3), mtCOI (4.4% divergence) and EF1α (1.0% divergence), with two divergent clades corresponding to EPR and Pacific-Antarctic Ridge (PAR, i.e., 31°S-38°S) populations, respectively. None of the three other nuclear genes (i.e., SAHH, Lyso, Sulfo1) were divergent across the Easter Microplate (Fig. 2, 3). Conversely, networks and geographic distribution of alleles along the ridge (Fig. 2, 3) revealed a pronounced geographic differentiation along the EPR for these three nuclear genes between the 9°50′N-7°25′S and 14°S-21°33′S regions in concordance with mtCOI, but not EF1α.

Nuclear gene networks exhibited two clades separated by a pronounced net divergence (Fig. 2). The SAHH gene revealed the presence of two clades (2% divergence) well established from each part of the previously suggested 7°25′S-14°S break (Fig. 2, Fig. 3, [17]). SAHH clade 1 contains the sequences from most individuals sampled at 9°50′N or 7°25′S whereas clade 2 corresponds to individuals sampled only from the southern EPR sites. Two 0.3%-divergent clades were also found using the Lyso gene but are more difficult to attribute to a particular geographic area (Fig. 2, 3). However, a 1-bp deletion was only found in the intron of sequences sampled in the southern EPR sites below the latitude of 7°25′S (Fig. 3), indicating that the 7°25′S–14°S barrier is playing a role in impeding the spread of new mutations. The Sulfo1 gene also displayed a clear geographic structure between north and south of the barrier with a 0.5% divergence between the two clades (Fig. 2).

### Statistical evidence for a 7°25′S–14°S barrier

The Monmonier analysis identified a barrier between 7°25′S and 14°S for all tested loci (Fig. 3) and *φ_st_* values were significantly different from zero for all sampled genes across this barrier (Table 2). An Isolation with Migration (IMa2) analysis was performed between the southern and northern populations across this barrier. The marginal posterior probability distribution of migration rate across the barrier overlapped with zero, indicating that the absence of migration cannot be ruled out. If present, migration across this barrier could have occurred in both directions, possibly slightly orientated from north to south (Table 3). The estimated effective population sizes of the present populations were both greater than the ancestral (*N_A_*) population size (Table 3), indicating that present-day populations of the vent mussel may be expanding, possibly at a higher rate in the south (*N_N_* being slightly higher than *N_S_*). However, expansion cannot be confirmed because of the overlap range of Highest Posterior Density among north, south and ancestral populations.

**Table 2:** Summary statistics of nucleotide polymorphism according to locality for nuclear genes and mitochondrial gene.

Locus	Locality	*h*	*S*	*θ_W_* ×100	*π_n _*×100	*φ_st_*
Sulfo1						0.474 ^***^
	9°50′N	4	3	0.511	0.617	
	7°25′S	9	12	1.674	1.196	
	14°S	3	4	0.613	0.309	
	17°25′S	5	6	0.920	0.463	
	17°35′S	5	5	0.767	0.386	
	18°33′S	5	4	0.613	0.379	
	21°33′S	4	4	0.714	0.546	
	38°S	1	-	-	-	
Lyso						0.108 ^***^
	9°50′N	11	24	0.665	0.692	
	7°25′S	18	23	0.534	0.592	
	14°S	10	30	0.766	0.566	
	17°25′S	9	29	0.832	0.568	
	17°35′S	11	25	0.625	0.415	
	18°33′S	10	26	0.699	0.579	
	21°33′S	13	34	0.868	0.542	
	38°S	2	7	-	-	
SAHH						0.717^***^
	9°50′N	6	18	0.918	0.893	
	7°25′S	6	16	0.494	0.687	
	14°S	10	34	1.479	1.140	
	17°25′S	9	41	1.649	0.955	
	17°35′S	8	11	0.439	0.341	
	18°33′S	8	8	0.344	0.270	
	21°33′S	17	36	1.270	0.594	
	38°S	2	6	-	-	
EF1α						0.033^NS^
	9°50′N	1	0	-	-	
	7°25′S	3	2	0.101	0.030	
	21°33′S	6	12	0.699	0.395	
	38°S	1	-	-	-	

Locus	Locality	*h*	*S*	*θ_W_* ×100	*π_n ×_*100	*φ_st_*
mtCOI						0.280^***^
	GMB	7	11	0.700	0.749	
	GRG	6	11	0.700	0.612	
	13°N	4	8	0.509	0.624	
	11°N	6	11	0.700	0.612	
	9°50′N	16	18	0.751	0.687	
	7°25′S	21	22	0.907	0.715	
	11°S	6	8	0.509	0.682	
	14°S	14	11	0.534	0.294	
	17°25′S	8	12	0.553	0.366	
	17°35′S	24	27	1.113	0.345	
	18°33′S	14	13	0.631	0.286	
	21°33′S	12	10	0.499	0.266	
	31°S	8	9	0.573	0.533	
	32°S	7	9	0.573	0.533	
	38°S	2	4	-	-	

*h*, number of different alleles across individuals; *S*, number of segregating sites; *θ_W_*, Watterson's theta; *π_n_*, nucleotide diversity. *φ_st_* values correspond to levels of differentiation between populations from 9°N-7°25′S and populations from 14°S-21°33′S. ***, P value < 0.001; NS, P value > 0.05. Sulfo 1, Sulfotransferase paralogue 1; Lyso, Lysozyme; SAHH, S-Adenosyl Homocysteine Hydrolase; EF1α, Elongation Factor 1α; mtCOI, cytochrome oxidase I.

**Table 3:** IMa2 estimates and the 95% Highest Posterior Density (HPD) intervals of migration and demographic parameters across the 7°25′S-14°S barrier to gene flow.

	*θ_N_*	*θ_S_*	*θ_A_*	*m_N-S_*	*m_S-N_*	*N_N_*	*N_S_*	*N_A_*	*M_N-S_*	*M_S-N_*
Estimate	5.125	5.975	3.575	0.373	0.003	642647	749232	448285	1.555	0.057
L-HPD	2.825	3.575	0.775	0.013	0.000	354239	448285	97181	0.000	0.000
H-HPD	11.720	44.380	41.230	0.989	1.103	1470250	5564379	5169386	11.010	3.343

L-HPD, lower 95% Highest Posterior Density; H-HPD, higher 95% Highest Posterior Density. *θ*, demographic parameter estimated by IMa2. *N*, calibration of *θ* in number of individuals. *m*, migration parameters estimated by IMa2. *M*, calibration of *m* in number of individuals. *_N_*, north of the barrier; *_S_*, south of the barrier; *_A_*, ancestral population; forward in time, *_N-S_*, migration from north to south; *_S-N_*, migration from south to north.

### Gene divergence homogeneity across the barrier

**Figure 4:**
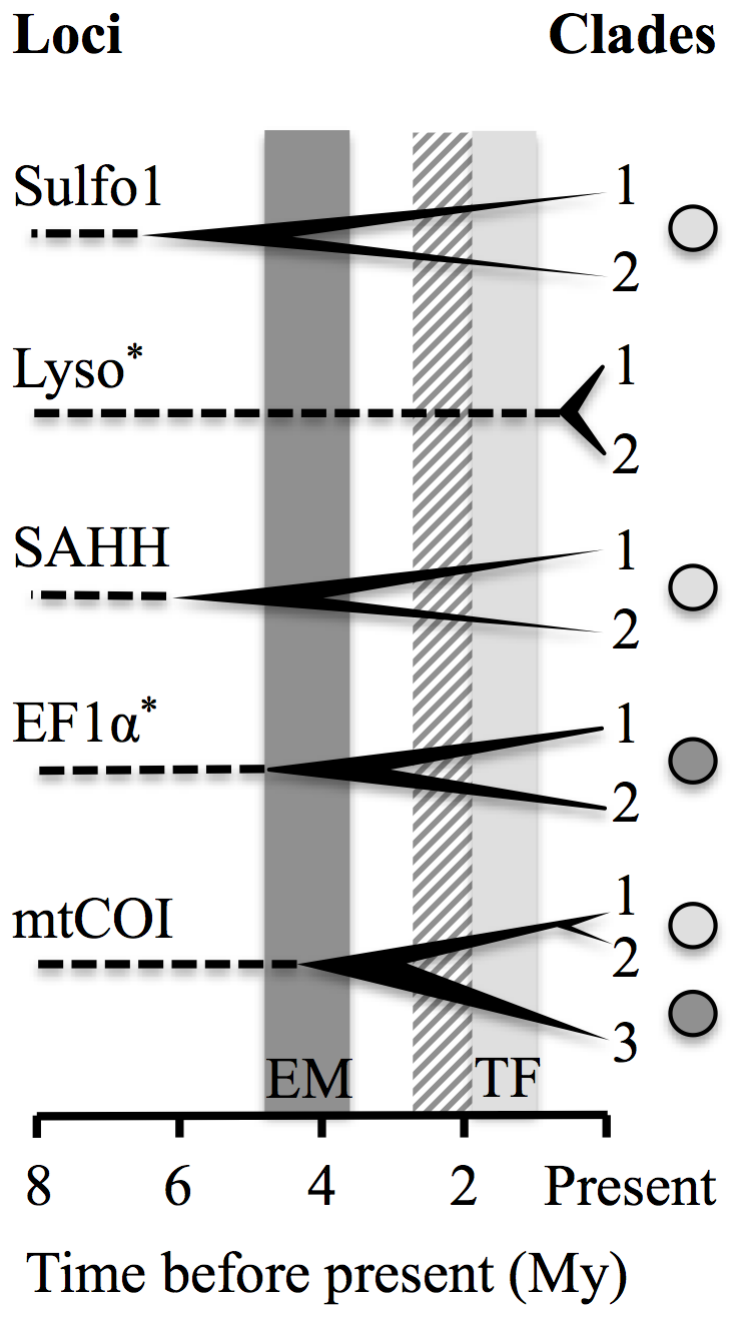
Bifurcated trees showing the correspondence between divergence times between sister clades and times of geological formations. Clades correspond in those identified in Fig. 2. Grey boxes represent the estimated time of the two barriers: EM, time since the first offsetting of overlapping faults leading to the Easter Microplate (dark grey); TF, time since the formation of Gofar/Discovery transform faults at 7°25′S–14°S latitude (light grey). Striped box represents the estimated time elapsed since the junction between the Pacific Antarctic Ridge and the East Pacific Rise. Grey circles correspond to the geographic position at which the divergence is observed, EM: dark grey and TF: light grey. Dashed lines represent the roots of the trees with the outgroup and calibration point (i.e. *Bathymodiolus azoricus*, Mid-Atlantic Ridge, about 8–12 Mya). Intervals of estimated divergence, due to the range of the calibration point date, are represented by horizontal thickness of tree nodes. Sulfo1, Sulfotranferase paralogous gene 1; Lyso, Lysozyme; SAHH, S-Adenosyl Homocysteine Hydrolase; mtCOI, mitochondrial cytochrome oxidase 1; EF1α, Elongation Factor 1α. ^*^Lysozyme and EF1α estimates of divergence are indicated but have to be interpreted with caution because these loci did not follow a strict molecular clock.

Under the verified assumption of a molecular clock (BEAST analysis) and using a 8–12 Mya calibration time obtained from the splitting of *B. thermophilus* and *B. azoricus* across the Isthmus of Panama, divergence times between clades 1 and 2 were estimated at 0.6–0.9 Mya for mtCOI, conforming to geological estimates of the ages of the transform faults in the 7°25′S–14°S area (Fig. 4). In contrast, divergence times between mtCOI clades 2 and 3 (3.0–4.5 Mya) as well as between Sulfo1 (4.4–6.6 Mya) and between SAHH (4.0–6.1 Mya) clades conformed to geological estimates for Easter Microplate formation (Fig. 4). Divergence times between clades for the Lyso (0.4–0.6 Mya) and EF1α (3.1–4.7 Mya) genes have to be interpreted with caution because of mutation rate heterogeneity among clades (departure from a strict molecular clock).

The multi-locus HKA test showed a significant departure from neutral evolution (P < 0.02) among genes within clade 1 (no departure among genes within clade 2), indicating that at least one gene could be under selection. Sulfo1 displayed the highest divergence to polymorphism deviation when compared to other genes. The removal of Sulfo1 (possible outlier) from the dataset led the HKA test to refit the model of neutral evolution (P > 0.31). This gene displayed a polymorphism/divergence ratio of 8.66: a value that is more than eight times greater than expected (1.05) with a very low divergence between *B. thermophilus* and *B. azoricus*, indicative of balancing or strong purifying selection.

### Detection of introgressed individuals using the SAHH gene

**Figure 5:**
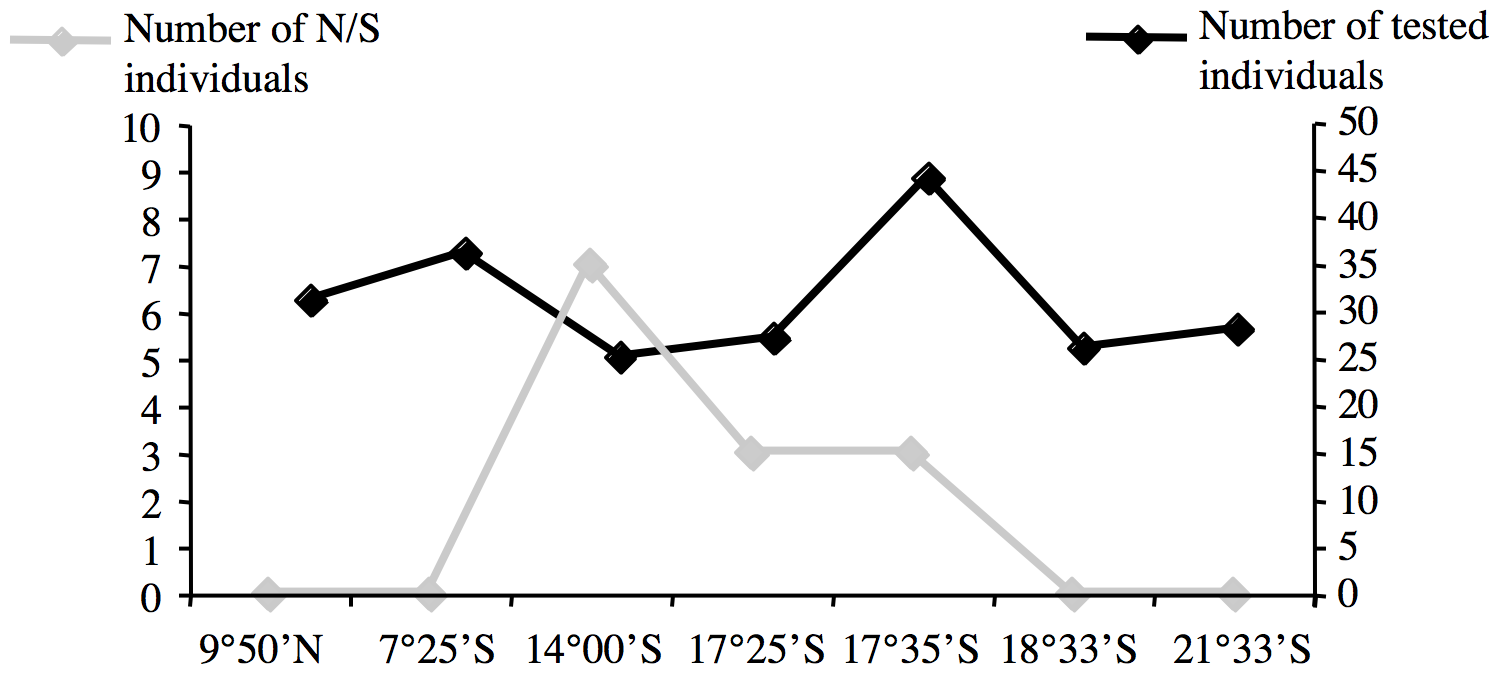
Localisation of individuals with both north and south type alleles in SAHH using RFLP analyses. N/S individuals, individuals that present one allele from the northern clade (blue clade in Figure 2) and one from the southern clade (yellow clade in Figure 2). Left Y-axis is the number of N/S individuals. Right Y-axis is the total number of individuals.

The RFLP analysis of the SAHH marker revealed that only 13 individuals south of the 7°25′S–14°S barrier displayed at least one nuclear allele corresponding to the northern clade. Except for one individual showing two northern-type alleles in the southern populations, all of these individuals possessed one allele of each clade. These N/S individuals were mainly located at 14°S and decreased abruptly in frequency further south (Fig. 5). No N/S individual was detected at 7°25′S or further north. N/S individuals of SAHH had Sulfo1 alleles from clade 2 only but they were not associated with a specific mtCOI clade: the SAHH northern-type allele being found in association with haplotypes from both the mtCOI clades 1 and 2, indicating that these individuals are most probably introgressed rather than first generation hybrids which should result in cyto-nuclear disequilibrium [45], [46].

## Discussion

The unpredictable nature of fluid circulation and the high level of fragmentation found at deep-sea hydrothermal vents should result in species with high dispersal capabilities in order to promote long-distance (re)colonization of new vent sites [47]. In accordance with this expectation, most species from the genus *Bathymodiolus* have high dispersal capabilities [20], [21], [22]. Data from *B. thermophilus* supports this hypothesis in showing a complete lack of differentiation at allozyme markers between the northern EPR (13°N, 11°N, 9°N) and the Galapagos sites [48] (but see [49]). Having high dispersal capabilities, however, does not necessarily imply a lack of geographic structure because gene flow is likely impacted by physical barriers to dispersal, great distances without suitable habitat and/or hybridization fronts [23].

Previous and present works strongly support the hypothesis that the population structure of *Bathymodiolus thermophilus* is impacted by two barriers to gene flow along the EPR: the so-called Easter Microplate barrier ([15], [24], present manuscript) and the 7°25′S–14°S barrier. This 7°25′S–14°S barrier was first suggested by Plouviez *et al.* [17], but then challenged by Audzijonyte & Vrijenhoek [25] who proposed that frequency changes in the two most divergent mtCOI clades found in the mussel populations along the EPR were most likely due to isolation-by-distance (Mantel Spearman test performed on the Won *et al.*'s dataset found significant [15]) and not an actual barrier. Johnson *et al.*'s study [24], together with our present work, refutes the hypothesis that isolation-by-distance alone might be responsible for the genetic patterns observed along the EPR, instead these studies suggest that a tension zone across the Easter Microplate might explain the clinal distribution of alleles. Johnson *et al.* [24] proposed the occurrence of a hybrid zone at 23°S and confirmed that alleles from the Pacific-Antarctic mussel *B. antarcticus* were likely to introgress in the South EPR populations of *B. thermophilus* but did not discuss the possible presence of a barrier to gene flow further north (7°25′–14°S).

In the present manuscript, we confirm the existence of such a barrier for the deep-sea mussel and argue that the observed positive correlation of *φ_st_* with geographic distances at the mtCOI locus (significant Mantel Spearman tests) likely reflects the occurrence of a tension zone that originated at the Easter Microplate (as proposed by Johnson *et al.* [24]), but which subsequently moved northward and became captured by a second area of restricted larval exchanges (responsible for the cline), the 7°25′S–14°S barrier to gene flow. As for many genetic barriers it is semipermeable and affects loci differentially.

### Genetic differentiation of B. thermophilus is triggered by the recent formation of a series of transform faults at 7°25′S–14°S

The Monmonier analysis and coalescence analyses statistically detected the presence of a barrier to gene flow at 7°25′S–14°S using nuclear and COI genes. Sulfo1 and SAHH loci both showed two sets of divergent alleles distributed in the 9°50′N–7°25′S and 14°S–21°33′S regions, with an almost reciprocal monophyly. The gene encoding Lyso exhibited two sets of divergent alleles that were geographically interspersed over the whole EPR and PAR, indicating that they were able to cross both the Easter Microplate and the 7°25′S–14°S barriers. However, the 1-bp deletion allele found at a high frequency in populations from 14°S-21°33′S was not observed from 9°50′S to 7°25′S nor within individuals from 38°S. The sample size (2 individuals) is far too low to exclude the absence of this deletion at 38°S. The absence of the deletion in the 9°50′N and 7°25′S individuals, however, strongly suggests that the spread of this new deletion has been blocked since its first occurrence in the southern populations (at least across the 7°25′S–14°S barrier). This deletion is however old enough to provide recombinant alleles at a high frequency between the two Lyso divergent clades, as the deletion is present at the tip of both divergent clades (see Fig. 2). The geographic distribution of this deletion on the Lyso gene suggests that this newly-formed barrier is now impermeable or only sporadically permeable to gene flow. The multigenic estimation of migration rates using a Bayesian approach and the mitochondrial and nuclear sequences (IMa2) also fit the relative isolation of mussels located at 7°25′S or 9°50′N when compared to the more southern populations. The IMa2 analysis indeed showed the absence or the very limited number of mussel migrants across the 7°25′S–14°S barrier. Although sequences of *B. thermophilus* from 11°S were not available for the other genes, we hypothesize this barrier is located, more precisely, between 11°S and 14°S based on haplotype frequencies of the mtCOI gene (*φ_st_*).

The barrier was however not sufficient to create an allele frequency shift at allozyme markers, highlighting an apparent discrepancy among marker types (allozymes vs. DNA sequences). Discrepancies in the genetic differentiation observed with allozyme and DNA markers have regularly been suggested to result from selection at allozyme loci, sometimes claimed to be under balancing selection [50], [51] and sometimes under disruptive selection [52], [53]. However, subsequent analyses often refute the hypothesis of selection on allozymes by demonstrating insufficient sampling [54], [55]. Here, EF1α is sufficient to show that a low level of differentiation is observed at a DNA marker in the area sampled for allozyme analysis. Furthermore, Johnson *et al.* [24] did not observe any genetic differentiation north of the Easter Microplate at the three DNA markers that are not in common between the two surveys. We conclude that there is no real discrepancy between the two categories of markers and that the few allozyme loci analyzed simply fell in genomic regions unlinked to isolation genes. These loci are thus able to cross the barrier freely and to organize themselves according to geographic distance, producing an IBD pattern. Moreover, the barrier may not be detectable on some of the allozyme loci (*Mpi*, *Odh*, *Mdh 2* and *Hk)* because of their low level of polymorphism.

Geologic and hydrodynamic conditions from the equator to 15°S are consistent with limited gene flow observed for multiple species between north and south EPR [3], [15], [16], [17]. In terms of geology, dispersal in this region can be impeded by the equatorial triple junction between the EPR and the Galapagos Rift, as well as the Gofar/Discovery multiple transform fault complex near 4°S [56], [57], [58]. When looking at local hydrodynamism in the region, surveys of He-3 plumes produced by the venting activity along the EPR also indicated the occurrence of a westward flow centered at 15°S [59], [60]. This flow, possibly linked to the anticyclonic circulation of water masses in the eastern Pacific [61], creates strong cross-axis currents able to produce a hydrodynamic barrier at these latitudes. The co-occurrence of these geologic and hydrodynamic features represents ideal conditions for the establishment of new barriers to gene flow.

### A dynamic effect of a couple of physical barriers to gene flow

Based on tectonic plate history, the Easter Microplate barrier is older than the 7°25′S–14°S barrier. The Easter Microplate originated from the progressive offsetting of the Pacific-Antarctic Ridge (PAR) along with the ‘old’ EPR (Nazca Pacific spreading centre) about 3.88 Mya (anomaly 3). This offsetting progressively expanded with the formation of an overlapping spreading center (OSC: see [62]). Ridge offsetting is known to have a profound impact on gene flow [17], [63], [64] and to isolate populations from each other. In the specific case of the Easter Microplate, species divergence time was thus expected to coincide with (or to be slightly older than) anomaly 3 (i.e., 3.88 Mya) at the Easter Microplate barrier. In comparison, transform faults responsible for offsetting the ridge axis between 9°N and 17°S initiated 1-2 Mya [56], [57], [58]. If the two geographical barriers were both impermeable to dispersal since their occurrence, one would expect to find reciprocal monophylies for nearly all genes, with a greater divergence, at the older barrier (i.e., the Easter Microplate barrier) and incomplete lineage sorting at the most recent barrier (i.e., the 7°25′S–14°S barrier).

Contrasting results among genes across the Easter Microplate are in accordance with the presence of a tension zone at the latitude of Easter Island as proposed by Johnson *et al.* [24] who studied the PAC/EPR population connectivity between 21°S to 38°S in more detail. The present results on mtCOI integrating both Won *et al.* [15] and Plouviez *et al.* [17] datasets are consistent with geological evidence, indicating that gene flow is either blocked or greatly impeded by both the Easter Microplate and the 7°25′S–14°S barriers. MtCOI haplotypes indeed show a reciprocal monophyly with a 2.1–4.3 Mya divergence time across the Easter Microplate [15], while populations display only haplotype frequency differences across the 7°25′S and 14°S barrier (significant φ*_st_*, [17] and Table 2). Absence of detectable differentiation on the EF1α gene across the more recent 7°25′S–14°S barrier (Table 2) while populations were divergent across the older Easter Microplate barrier was expected considering nuclear genes generally evolve slower than mitochondrial genes. In contrast, the other nuclear gene structure did not fit this expectation; showing no differentiation across the Easter Microplate, whereas two of them (SAHH, Sulfo1) displayed clear but unexpected patterns of reciprocal monophyly across the more recent barrier (7°25′S–14°S). Such an inconsistency among genes might be explained by the presence of a tension zone extending further north toward 7°25′S associated with the relaxation of the Easter Microplate barrier.

The hypothesis of a relaxation of the barrier at the Easter Microplate is also supported by the geological formation of the microplate itself. If initiated by the offsetting of the PAR and ‘old’ EPR, the plate formation was achieved when the ends of the two expanding ridges joined together following a clockwise rotation with the setting up of insular volcanic arcs (Orongo and Pito rifts) about 2.5–1.75 Mya (end of anomaly 2a: see [65]). This junction between the PAR and EPR is likely to have provided stepping-stones for episodic bursts of larvae between the PAR and the southern EPR (using vents located on active seamounts: e.g., the Pito Seamount [66]).

We propose that the progressive ridge-offsetting at 7°25′–14°S played an important role in enhancing barriers to dispersal by efficiently trapping endogenously-induced genetic clines associated with the reconnection of *B. thermophilus* and *B. antarcticus*, which could have subsequently escaped from the relaxing barrier at the Easter Microplate. Divergence times between clades for the SAHH and Sulfo1 nuclear genes across the 7°25′S–14°S barrier (4–6.6 Mya) were similar to those estimated at the Easter Microplate boundary between the PAC and SEPR clades of mtCOI and EF1α. This convergence in divergence times found at two distinct spatial locations, as well as the fact that nuclear mutation rates should be lower than that of the mtCOI (as found in the Atlantic *Bathymodiolus sp.* [23]) fits this “dual relaxing/enhancing barriers” hypothesis. Indeed, the fixation of alleles in the two observed-divergent clades associated with the SAHH and Sulfo1 loci (and possibly alleles from the Lyso gene as well) might have been initiated by the separation of populations during the Easter Microplate formation before migrating northward and being trapped by the emerging 7°25′S–14°S barrier.

To conclude, we have shown that the clinal distribution of mtCOI haplotypes along the EPR was in fact due to highly dynamic historical/geological processes. A system of two genetic barriers to gene flow, in which one would be progressively relaxed (i.e., Easter Microplate) and the other enforced (i.e., 7°25′S–14°S transform faults), can explain the discordant distribution of genetic clines. A volcanic arc joining PAR and EPR probably played an important role in setting up of a secondary contact zone (and subsequent northward spreading of alleles/genetic clines) between individuals across the Easter Microplate. The proposed relaxation/enforcement scenario of barriers is consistent with the occurrence of a biogeographic transition zone along the southern EPR (resulting from the overlap of the North-EPR and the South-Easter Microplate biogeographic provinces) to explain the high diversity of vent fauna species observed between 17°25′S and 21°33′S [67]. Studies on motion of hybrid/tension zones are in their infancy (but see e.g., [12], [68], [69], [70]). Tension zone movement is expected theoretically although they are expected to move toward areas of lower population density [5], [71]. Evidence for the movement of hybrid zones has been obtained directly following known tension zones over time (e.g., [68]) or indirectly using molecular and/or phenotypic traits (e.g., [70], review in [69]). Theoretical analyses have demonstrated the ability of low-migration areas (i.e., lack of suitable habitats/larval transport disruption) to trap genetic incompatibilities previously generated by other historical/ecological/biogeographical processes (see: [12]) and might apply to the case of *B. thermophilus*, which, in turn represents one of the first empirical observation of such theoretical predictions. The theory of genetic barriers and hybrid zones explains semi-permeable barriers to gene flow and the structure observed, possibly indicating selection against hybrids. Of course one could also speculate that different loci respond to different ecological pressures at the two positions. However, not only are the possible ecological differences unidentified but getting by chance a series of outlier loci (or hichhicked introns) under differential ecological selection out of four genes is highly unlikely. This therefore suggests that allele frequency shifts are linked to tension zones and are thus able to move. Together with what we know of the geological history of the EPR, the hypothesis of an interchange of clines from the southern barrier to the northern barrier explains the geographic discordance of cline positions.

## Supporting Information

Table S1 GenBank accession numbers of each unique sequence and their geographic distribution. Numbers correspond to the number of individuals having the accession number in a given population. For example, accession numbers KC858658 through KC858662 all have a single individuals recovered in the 9°50′N population.
(DOCX)

## References

[1] Kimura M, Weiss GH (1964) The stepping stone model of population structure and the decrease of genetic correlation with distance. Genetics 49: 561–576. 17248204 10.1093/genetics/49.4.561 PMC1210594

[2] Fontaine MC, Baird SJE, Piry S, Ray N, Tolley KA, et al (2007) Rise of oceanographic barriers in continuous populations of a cetacean: the genetic structure of harbour porpoises in Old World waters. BMC Biol 5: 30. 17651495 10.1186/1741-7007-5-30 PMC1971045

[3] Plouviez S, Le Guen D, Lecompte O, Lallier FH, Jollivet D (2010) Determining gene flow and influence of selection across the equatorial barrier of the East Pacific Rise in the tube-dwelling polychaete *Alvinella pompejana* . BMC Evol Biol 10: 220. 20663123 10.1186/1471-2148-10-220 PMC2924869

[4] Hewitt GM (1975) A sex chromosome hybrid zone in the grasshopper *Podisma pedestris* (Orthoptera: Acrididae). Heredity 35: 375–387. 1061710 10.1038/hdy.1975.108

[5] Barton NH (1979) The dynamics of hybrid zones. Heredity 43: 341–359.

[6] Slatkin M (1973) Gene flow and selection in a cline. Genetics 75: 733–756. 4778791 10.1093/genetics/75.4.733 PMC1213045

[7] Barton NH, Hewitt GM (1985) Analysis of hybrid zones. Annu Rev Ecol Evol Syst 16: 113–148.

[8] Castric V, Bernatchez L (2003) The rise and fall of isolation by distance in the anadromous brook charr (*Salvelinus fontinalis* Mitchill). Genetics 163: 983–996. 12663537 10.1093/genetics/163.3.983 PMC1462472

[9] Lewontin RC, Krakauer J (1973) Distribution of gene frequency as a test of the theory of the selective neutrality of polymorphism. Genetics 74: 175–195. 4711903 10.1093/genetics/74.1.175 PMC1212935

[10] Olson MS, Levsen N (2012) Classic clover cline clues. Mol Ecol 21: 2315–2317. 22548253 10.1111/j.1365-294X.2012.05503.x

[11] Hewitt GM (1988) Hybrid zones, natural laboratories for evolutionary studies. Trends Ecol Evol 3: 158–167. 21227192 10.1016/0169-5347(88)90033-X

[12] Bierne N, Welch J, Loire E, Bonhomme F, David P (2011) The coupling hypothesis: why genome scans may fail to map adaptation genes. Mol Ecol 20: 2044–2072. 21476991 10.1111/j.1365-294X.2011.05080.x

[13] Vrijenhoek RC (1997) Gene flow and genetic diversity in naturally fragmented metapopulations of deep-sea hydrothermal vent animals. J Hered 88: 285–293. 9262010 10.1093/oxfordjournals.jhered.a023106

[14] Jollivet D, Chevaldonné P, Planque B (1999) Hydrothermal-vent alvinellid polychaete dispersal in the eastern Pacific. 2. A metapopulation model based on habitat shifts. Evolution 53: 1128–1142. 28565536 10.1111/j.1558-5646.1999.tb04527.x

[15] Won Y, Young CR, Lutz RA, Vrijenhoek RC (2003) Dispersal barriers and isolation among deep-sea mussel populations (Mytilidae: *Bathymodiolus*) from eastern Pacific hydrothermal vents. Mol Ecol 12: 169–184. 12492886 10.1046/j.1365-294x.2003.01726.x

[16] Hurtado L, Lutz R, Vrijenhoek RC (2004) Distinct patterns of genetic differentiation among annelids of eastern Pacific hydrothermal vents. Mol Ecol 13: 2603–2615. 15315674 10.1111/j.1365-294X.2004.02287.x

[17] Plouviez S, Shank TM, Faure B, Daguin-Thiébaut C, Viard F, et al (2009) Comparative phylogeography among hydrothermal vent species along the East Pacific Rise reveals vicariant processes and population expansion in the South. Mol Ecol 18: 3903–3917. 19709370 10.1111/j.1365-294X.2009.04325.x

[18] Remington CL (1968) Suture-zones of hybrid interaction between recently joined biotas. Evol Biol 2: 321–428.

[19] Lutz RA, Jablonski D, Turner RD (1984) Larval development and dispersal at deep-sea hydrothermal vents. Science 226: 1451–1454. 17789002 10.1126/science.226.4681.1451

[20] Arellano SM, Young CM (2009) Spawning, development and the duration of larval life in a deep-sea cold-seep mussel. Biol Bull 216: 149–162. 19366926 10.1086/BBLv216n2p149

[21] Olu-Le Roy L, von Cosel R, Hourdez S, Carney SL, Jollivet D (2007) Amphi-Atlantic cold-seep *Bathymodiolus* species complexes across the equatorial belt. Deep Sea Res Part 1 54: 1890–1911.

[22] Carney SL, Formica MI, Divatia H, Nelson K, Fisher CR, et al (2006) Population structure of the mussel “*Bathymodiolus*” *childressi* from Gulf of Mexico hydrocarbon seeps. Deep Sea Res Part 1 53: 1061–1072.

[23] Faure B, Jollivet D, Tanguy A, Bonhomme F, Bierne N (2009) Speciation in the deep sea: multi-locus analysis of divergence and gene flow between two hybridizing species of hydrothermal vent mussels. PLoS One 4: e6485 doi:10.1371/journal.pone.0006485 19649261 10.1371/journal.pone.0006485 PMC2715857

[24] Johnson SB, Won Y-J, Harvey JBJ, Vrijenhoek RC (2013) A hybrid zone between *Bathymodiolus* mussel lineages from eastern Pacific hydrothermal vents. BMC Evol Biol 13: 21. 23347448 10.1186/1471-2148-13-21 PMC3740784

[25] Audzijonyte A, Vrijenhoek RC (2010) When gaps are really gaps: statistical phylogeography of hydrothermal vent invertebrates. Evolution 64: 2369–2384. 20298432 10.1111/j.1558-5646.2010.00987.x

[26] Boutet I, Tanguy A, Le Guen D, Piccino P, Hourdez, et al (2009) Global depression in gene expression as a response to rapid thermal changes in vent mussels. Proc R Soc Lond B 276: 3071–3079. 10.1098/rspb.2009.0503 PMC2817120 19515664

[27] Belkhir K, Borsa P, Chikhi L, Raufaste N, Bonhomme F (2004) Genetix 4.05, logiciel sous Windows TM pour la génétique des populations. Laboratoire Génome, Populations, Interactions, Adaptations, UMR 5000, Université de Montpellier 2. Available at: www.genetix.univ-montp2.fr/genetix/genetix.htm

[28] Weir B, Cockerham C (1984) Estimating F-statistics for the analysis of population structure. Evolution 38: 1358–1370. 28563791 10.1111/j.1558-5646.1984.tb05657.x

[29] Raymond M, Rousset F (1995) GENEPOP, version 1.2: population genetics software for exact tests and ecumenicism. J Hered 86: 248–249.

[30] Jolly MT, Viard F, Gentil F, Thiébaut E, Jollivet D (2006) Comparative phylogeography of two coastal polychaete tubeworms in the Northeast Atlantic supports shared history and vicariant events. Mol Ecol 15: 1841–1855. 16689902 10.1111/j.1365-294X.2006.02910.x

[31] Bierne N, Tanguy A, Faure M, Faure B, David E, et al (2007) Mark-recapture cloning: a straightforward and cost-effective cloning method for population genetics of single copy nuclear DNA sequences in diploids. Mol Ecol Notes 7: 562–566.

[32] Faure B, Bierne N, Tanguy A, Bonhomme F, Jollivet D (2007) Evidence for a slightly deleterious effect of intron polymorphisms at the EF1α gene in the deep-sea hydrothermal vent bivalve *Bathymodiolus* . Gene 406: 99–107. 17707599 10.1016/j.gene.2007.06.025

[33] Thompson JD, Higgins DG, Gibson TJ (1994) CLUSTAL W: improving the sensitivity of progressive multiple sequence alignment through sequence weighting, position specific gap penalties and weight matrix choice. Nucleic Acids Res 22: 4673–4680. 7984417 10.1093/nar/22.22.4673 PMC308517

[34] Rozas J, Sanchez-DelBarrio JC, Messeguer X, Rozas R (2003) DnaSP, DNA polymorphism analyses by the coalescent and other methods. Bioinformatics 19: 2496–2497. 14668244 10.1093/bioinformatics/btg359

[35] Bandelt HJ, Forster P, Rohl A (1999) Median-joining networks for inferring intraspecific phylogenies. Mol Biol Evol 16: 37–48. 10331250 10.1093/oxfordjournals.molbev.a026036

[36] Drummond AJ, Rambaut A (2007) BEAST: Bayesian evolutionary analysis by sampling trees. BMC Evol Biol 7: 214. 17996036 10.1186/1471-2148-7-214 PMC2247476

[37] Kumar S, Balczarek KA, Lai Z-C (1996) Evolution of the hedgehog gene family. Genetics 142: 965–972. 8849902 10.1093/genetics/142.3.965 PMC1207033

[38] Burton KW, Ling H-F, O’Nions RK (1997) Closure of the Central American Isthmus and its effect on deep-water formation in the North Atlantic. Nature 386: 382–385.

[39] Stiller J, Rousset V, Pleijel F, Chevaldonné P, Vrijenhoek RC, et al (2013) Phylogeny, biogeography and systematics of hydrothermal vent and methane seep *Amphisamytha* (Ampharetidae, Annelida), with descriptions of three new species. Syst Biodivers 11: 35–65.

[40] Hudson RR, Kreitman M, Aguade M (1987) A test of neutral molecular evolution based on nucleotide data. Genetics 116: 153–159. 3110004 10.1093/genetics/116.1.153 PMC1203113

[41] McDonald JH, Kreitman M (1991) Adaptive protein evolution at the Adh locus in *Drosophila* . Nature 351: 652–654. 1904993 10.1038/351652a0

[42] Manni F, Guérard E, Heyer E (2004) Geographic patterns of (genetic, morphologic, linguistic) variation: how barriers can be detected by “Monmonier's algorithm”. Hum Biol 76: 173–190. 15359530 10.1353/hub.2004.0034

[43] Hudson RR, Slatkin M, Maddison WP (1992) Estimation of levels of gene flow from DNA-sequence data. Genetics 132: 583–589. 1427045 10.1093/genetics/132.2.583 PMC1205159

[44] Hey J, Nielsen R (2007) Integration within the Felsenstein equation for improved Markov chain Monte Carlo methods in population genetics. Proc Nat Acad Sci USA 104: 2785–2790. 17301231 10.1073/pnas.0611164104 PMC1815259

[45] Won Y, Hallam SJ, O'mullan GD, Vrijenhoek RC (2003) Cytonuclear disequilibrium in a hybrid zone involving deep-sea hydrothermal vent mussels of the genus *Bathymodiolus* . Mol Ecol 12: 3185–3190. 14629398 10.1046/j.1365-294x.2003.01974.x

[46] Bierne N, Borsa P, Daguin C, Jollivet D, Viard F, et al (2003) Introgression patterns in the mosaic hybrid zone between *Mytilus edulis* and *M. galloprovincialis* . Mol Ecol 12: 447–461. 12535095 10.1046/j.1365-294x.2003.01730.x

[47] Mullineaux LS, Adams DK, Mills SW, Beaulieu SE (2010) Larvae from afar colonize deep-sea hydrothermal vents after a catastrophic eruption. Proc Natl Acad Sci USA 107: 7829–7834. 20385811 10.1073/pnas.0913187107 PMC2867905

[48] Craddock C, Hoeh WR, Lutz RA, Vrijenhoek RC (1995) Extensive gene flow among mytilid (*Bathymodiolus thermophilus*) populations from hydrothermal vents of the eastern Pacific. Mar Biol 124: 137–146.

[49] Grassle JF (1985) Genetic differentiation in populations of hydrothermal vent mussels (*Bathymodiolus thermophilus*) from the Galapagos Rift and 13°N on the East Pacific Rise. Bull Biol Soc Wash 6: 429–442.

[50] Karl SA, Avise JC (1992) Balancing selection at allozyme loci in oysters: implications from nuclear RFLPs. Science 256: 100–102. 1348870 10.1126/science.1348870

[51] Pogson GH, Mesa KA, Boutilier RG (1995) Genetic population structure and gene flow in the Atlantic cod *Gadus morhua*: a comparison of allozyme and nuclear RFLP loci. Genetics 139: 375–385. 7705638 10.1093/genetics/139.1.375 PMC1206334

[52] Lemaire C, Allegrucci G, Naciri M, Bahri-Sfar L, Kara H, et al (2000) Do discrepancies between microsatellite and allozyme variation reveal differential selection between sea and lagoon in the sea bass (*Dicentrarchus labrax*)? Mol Ecol 9: 457–467. 10736048 10.1046/j.1365-294x.2000.00884.x

[53] Riginos C, Sukhdeo K, Cunningham CW (2002) Evidence for selection at multiple allozyme loci across a mussel hybrid zone. Mol Biol Evol 19: 347–351. 11861894 10.1093/oxfordjournals.molbev.a004088

[54] McDonald JH, Verrelli BC, Geyer LB (1996) Lack of geographic variation in anonymous nuclear polymorphisms in the American oyster, *Crassostrea virginica* . Mol Biol Evol 13: 1114–1118. 8865664 10.1093/oxfordjournals.molbev.a025673

[55] Bierne N, Daguin C, Bonhomme F, David P, Borsa P (2003) Direct selection on allozymes is not required to explain heterogeneity among marker loci across a *Mytilus* hybrid zone. Mol Ecol 12: 2505–2510. 12919488 10.1046/j.1365-294x.2003.01936.x

[56] Kureth CL, Rea DK (1981) Large-scale oblique features in an active transform fault, the Wilkes fracture zone near 9°S on the East Pacific Rise. Mar Geophys Res 5: 119–137.

[57] Naar DF, Hey RN (1989) Speed limit for oceanic transform faults. Geology 17: 420–422.

[58] Francheteau J, Armijo R, Cheminee JL, Hekinian R, Lonsdale P, et al (1990) 1 Ma East Pacific Rise oceanic-crust and uppermost mantle exposed by rifting in Hess Deep (equatorial Pacific-ocean). Earth Planet Sci Lett 101: 281–295.

[59] Lupton JE, Craig H (1981) A major He-3 source at 15°S on the East Pacific Rise. Science 214: 13–18. 17802550 10.1126/science.214.4516.13

[60] Lupton JE (1998) Hydrothermal helium plumes in the Pacific ocean. J Geophys Res Oceans 103: 15853–15868.

[61] Fujio SZ, Imasato N (1991) Diagnostic calculation for circulation and water mass movement in the deep Pacific. J Geophys Res Oceans 96: 759–774.

[62] Tebbens SF, Cande SC (1997) Southeast Pacific tectonic evolution from Oligocene to present. J Geophys Res Solid Earth 102: 12601–12084.

[63] Johnson SB, Young CR, Jones WJ, Warén A, Vrijenhoek RC (2006) Migration, isolation, and speciation of hydrothermal vent limpets (Gastropoda; Lepetodrilidae) across the Blanco transform fault. Biol Bull 210: 140–157. 16641519 10.2307/4134603

[64] Young CR, Fujio S, Vrijenhoek RC (2008) Directional dispersal between mid-ocean ridges: deep-ocean circulation and gene flow in *Ridgeia piscesae* . Mol Ecol 17: 1718–1731. 18371015 10.1111/j.1365-294X.2008.03609.x

[65] Larson RL, Searle RC, Kleinrock MC, Schouten H, Bird RT, et al (1992) Roller-bearing tectonic evolution at the Juan Fernandez Microplate. Nature 356: 571–576.

[66] Naar DF, Hekinian R, Segonzac M, Francheteau J, Armijo R, et al (2004) Vigorous venting and biology at Pito seamount, Easter Microplate. Geophys Monograph Ser 148: 305–318.

[67] Matabos M, Plouviez S, Hourdez S, Desbruyères D, Legendre P, et al (2011) Faunal changes and geographic crypticism indicate the occurrence of biogeographic transition zone along the southern East Pacific Rise. J Biogeogr 38: 575–594.

[68] Dasmahapatra KK, Blum MJ, Aiello A, Hackwell S, Davies N, et al (2002) Inferences from a rapidly moving hybrid zone. Evolution 56: 741–753. 12038532 10.1111/j.0014-3820.2002.tb01385.x

[69] Buggs RJA (2007) Empirical study of hybrid zone movement. Heredity 99: 301–312. 17611495 10.1038/sj.hdy.6800997

[70] Gay L, Crochet P-A, Bell DA, Lenormand T (2008) Comparing clines on molecular and phenotypic traits in hybrid zones: a window on tension zone models. Evolution 62: 2789–2806. 18752618 10.1111/j.1558-5646.2008.00491.x

[71] Barton NH, Turelli M (2011) Spatial waves of advance with bistable dynamics: cytoplasmic and genetic analogues of Allee effects. Am Nat 178: 48–75 doi: 10.1086/661246 10.1086/661246 21828986

