# Supplementary material for: A New Barrier to Dispersal Trapped Old Genetic Clines That Escaped the Easter Microplate Tension Zone of the Pacific Vent Mussels
Source: PLoS One. 2013 Dec 2;8(12):e81555. doi: 10.1371/journal.pone.0081555 (PMC3846894; doi:10.1371/journal.pone.0081555)
Supplement: Table S1 — GenBank accession numbers of each unique sequence and their geographic distribution. Numbers correspond to the number of individuals having the accession number in a given population. For example, accession numbers KC858658 through KC858662 all have a single individuals recovered in the 9°50′N population. (DOCX) [file pone.0081555.s001.docx]

| Locus | Accession Number | 9°50’N | 7°25’S | 14°S | 17°25’S | 17°35’S | 18°33’S | 21°33’S | 38°S |
| --- | --- | --- | --- | --- | --- | --- | --- | --- | --- |
| SAHH | KC858658-62 | 1 |  |  |  |  |  |  |  |
|  | KC858663 | 1 | 4 | 1 |  |  |  |  |  |
|  | KC858664-78 |  | 1 |  |  |  |  |  |  |
|  | KC858679-81 |  | 2 |  |  |  |  |  |  |
|  | KC858682-89 |  |  | 1 |  |  |  |  |  |
|  | KC858690 |  |  | 1 | 1 | 2 |  | 1 |  |
|  | KC858691 |  |  | 1 |  | 1 |  |  | 1 |
|  | KC858692 |  |  | 1 |  |  | 2 | 2 |  |
|  | KC858693-701 |  |  |  | 1 |  |  |  |  |
|  | KC858702 |  |  |  | 1 |  | 1 |  |  |
|  | KC858703 |  |  |  |  | 1 | 1 |  |  |
|  | KC858704-09 |  |  |  |  | 1 |  |  |  |
|  | KC858710-16 |  |  |  |  |  | 1 |  |  |
|  | KC858717-32 |  |  |  |  |  |  | 1 |  |
|  | KC858733 |  |  |  |  |  |  |  | 1 |
| Lyso | KC858734-43 | 1 |  |  |  |  |  |  |  |
|  | KC858744 | 1 | 1 |  |  |  |  |  |  |
|  | KC858745-58 |  | 1 |  |  |  |  |  |  |
|  | KC858759 |  | 2 | 2 |  |  |  |  |  |
|  | KC858760 |  | 1 |  |  | 1 | 1 |  |  |
|  | KC858761 |  | 1 |  |  |  |  | 1 |  |
|  | KC858762-69 |  |  | 1 |  |  |  |  |  |
|  | KC858770 |  |  | 1 |  | 1 |  |  |  |
|  | KC858771 |  |  | 2 |  |  |  |  |  |
|  | KC858772 |  |  | 1 |  | 1 |  | 1 |  |
|  | KC858773-81 |  |  |  | 1 |  |  |  |  |
|  | KC858782 |  |  |  | 1 | 1 |  |  |  |
|  | KC858783-88 |  |  |  |  | 1 |  |  |  |
|  | KC858789 |  |  |  |  | 3 | 2 | 1 |  |
|  | KC858790 |  |  |  |  | 2 |  |  |  |
|  | KC858791-99 |  |  |  |  |  | 1 |  |  |
|  | KC858800-10 |  |  |  |  |  |  | 1 |  |
|  | KC858811-12 |  |  |  |  |  |  |  | 1 |

| Locus | Accession Number | 9°50’N | 7°25’S | 14°S | 17°25’S | 17°34’S | 18°33’S | 21°33’S | 38°S |
| --- | --- | --- | --- | --- | --- | --- | --- | --- | --- |
| Sulfo1 | KC858813 | 3 | 3 |  |  |  |  |  |  |
|  | KC858814 | 1 |  |  |  |  |  |  |  |
|  | KC858815 | 4 | 5 |  |  |  |  |  |  |
|  | KC858816 | 1 |  |  |  |  |  |  |  |
|  | KC858817-21 |  | 1 |  |  |  |  |  |  |
|  | KC858822 |  | 2 |  |  |  |  |  |  |
|  | KC858823 |  | 1 | 10 | 8 | 8 | 8 | 4 | 2 |
|  | KC858824-25 |  |  | 1 |  |  |  |  |  |
|  | KC858826-29 |  |  |  | 1 |  |  |  |  |
|  | KC858830-33 |  |  |  |  | 1 |  |  |  |
|  | KC858834 |  |  |  |  | 1 | 2 |  |  |
|  | KC858835-37 |  |  |  |  |  | 1 |  |  |
|  | KC858838-39 |  |  |  |  |  |  | 1 |  |
| EF1α | KC858840 | 4 | 23 |  |  |  |  | 11 |  |
|  | KC858841 |  | 1 |  |  |  |  | 1 |  |
|  | KC858842 |  | 1 |  |  |  |  |  |  |
|  | KC858843-45 |  |  |  |  |  |  | 1 |  |
|  | KC858846 |  |  |  |  |  |  | 1 | 2 |
